# Supplementary material for: Sequence-specific capture and concentration of viral RNA by type III CRISPR system enhances diagnostic
Source: Nat Commun. 2022 Dec 15;13:7762. doi: 10.1038/s41467-022-35445-5 (PMC9751510; doi:10.1038/s41467-022-35445-5)
Supplement: Supplementary file 2 — Supplementary Information [file 41467_2022_35445_MOESM2_ESM.pdf]

Supplementary Information for manuscript

# Sequence-specific capture and concentration of viral RNA by type III CRISPR system enhances diagnostic

Anna Nemudraia[1,3], Artem Nemudryi[1,3], Murat Buyukyoruk[1], Andrew M. Scherffius[1], Trevor Zahl[1], Tanner Wiegand[1], Shishir Pandey[1], Joseph E. Nichols[1], Laina Hall[1], Aidan McVey[1], Helen H Lee[1], Royce A. Wilkinson[1], Laura R. Snyder[2], Joshua D. Jones[2], Kristin S. Koutmou[2], Andrew Santiago-Frangos[1], and Blake Wiedenheft[1]

[1]Department of Microbiology and Cell Biology, Montana State University, Bozeman, MT 59717, USA

[2]Department of Chemistry, University of Michigan, Ann Arbor, MI 48105, USA

[3]These authors contributed equally: Anna Nemudraia, Artem Nemudryi

Corresponding authors: Blake Wiedenheft (bwiedenheft@gmail.com), Andrew Santiago-Frangos (andrew.santiagofrangos@gmail.com)

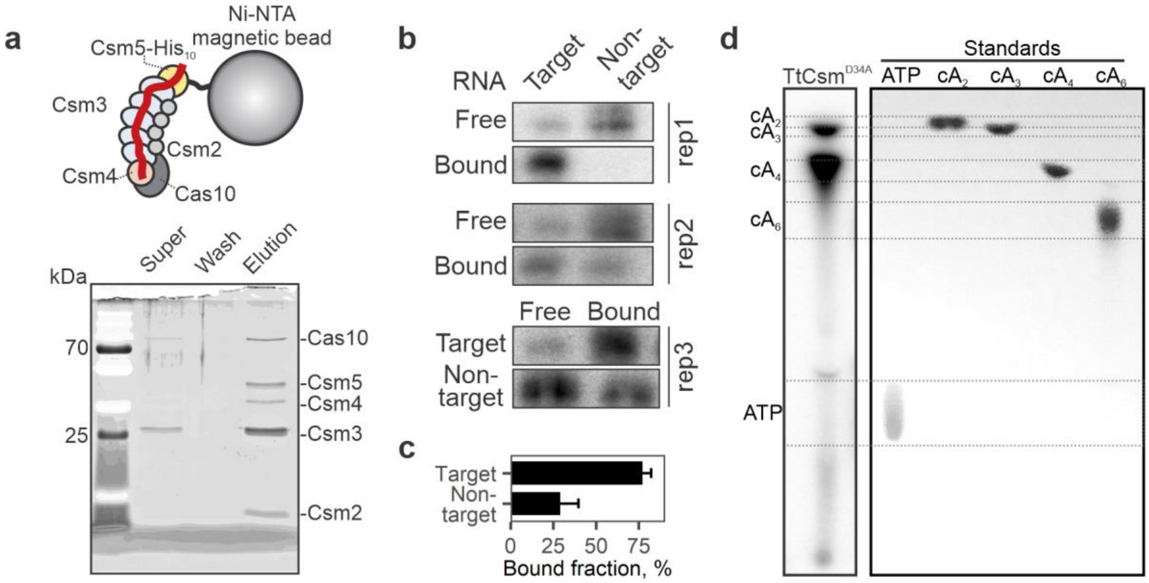


**Supplementary Figure 1: Type III CRISPR-based RNA concentration enhances detection. a** *Top panel:* schematic of TtCsm-complex bound to a Ni-NTA magnetic bead. *Bottom panel:* SDS-PAGE of TtCsm-complex after pull-down with Ni-NTA magnetic beads. After binding, beads were separated with a magnet, and supernatant ("Super") was decanted. The beads were washed once ("Wash"), and the protein was eluted using 250 mM imidazole ("Elution"). **b** Sequence-specific RNA enrichment with TtCsm$^{Csm3\text{-}D34A}$ complex was tested using $^{32}$P 5`-end labeled IVT RNA fragments. Target and non-target RNA fragments were mixed with TtCsm$^{Csm3\text{-}D34A)}$ complex, incubated at 65°C for 20 min and pulled-down using a Ni-NTA magnetic beads. After the magnetic pull-down, RNA was phenol-chloroform extracted from supernatants ("Free") and bead pellets ("Bound"). Extracted RNA was resolved using UREA-PAGE, exposed to a phosphor screen, and imaged on a Typhoon 5 imager (Amersham). Three replicates are shown. **c** RNA bands shown in **b** were quantified using ImageJ and the bound fraction was calculated for each replicate as follows, [bound/(bound + free)*100%]. Bars show mean of 3 replicates ± S.E.M.; **d** TtCsm$^{Csm3\text{-}D34A}$ complex produces $cA_3$ and $cA_4$. TtCsm$^{Csm3\text{-}D34A}$ complex (25 nM) was mixed with target RNA ($10^{11}$ copies/ul) to activate Cas10 subunit. Polymerization reaction was performed with α-$^{32}$P-ATP. Reaction products were phenol-chloroform extracted and resolved on the same thin-layer chromatography (TLC) plate with chemically synthesized standards. Radiolabeled products (left) were imaged with Typhoon 5 phosphor imager. Chemically synthesized standards ($cA_2$-$cA_6$) were visualized using UV shadowing (right).

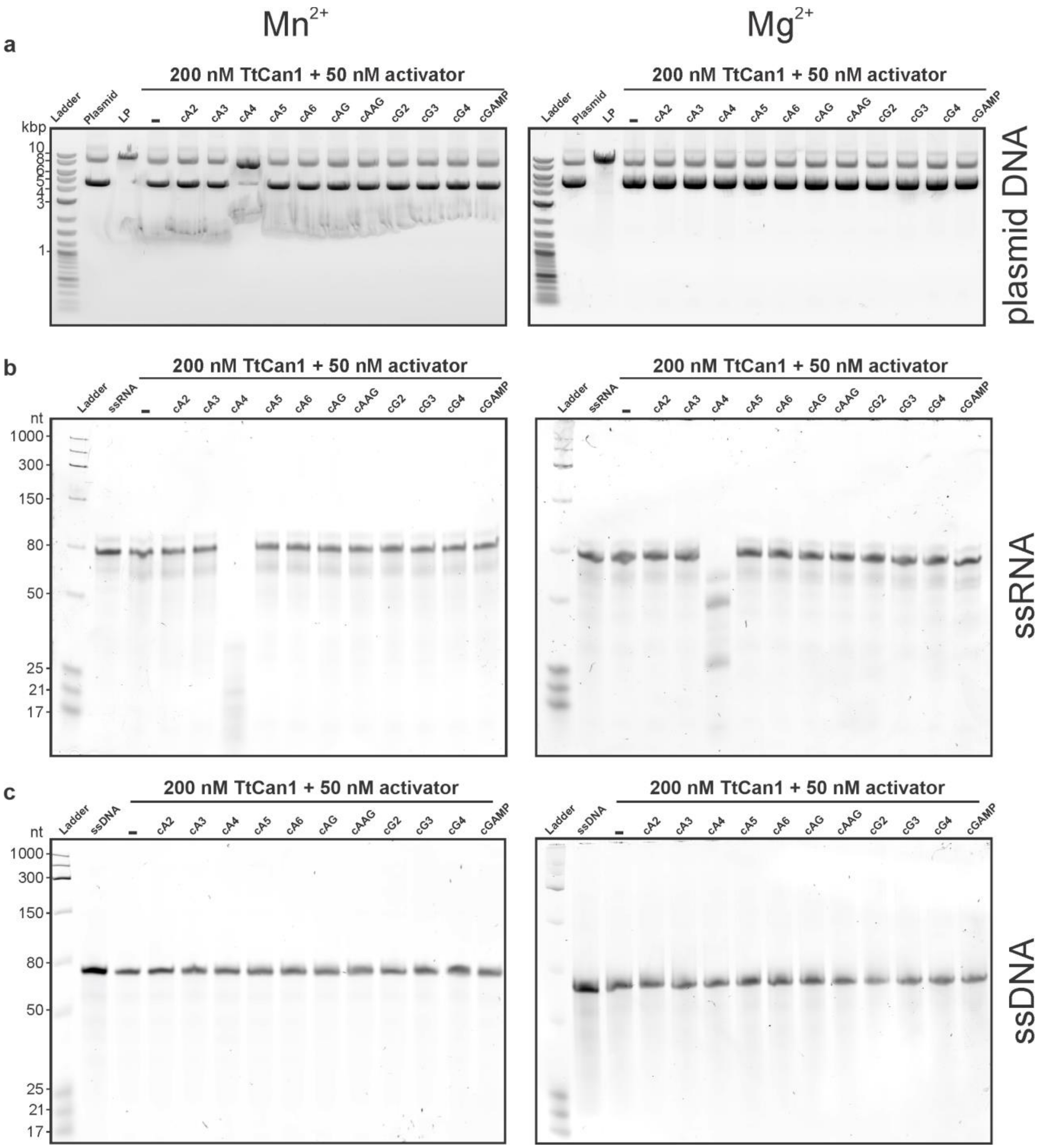


**Supplementary Figure 2: Biochemical characterization of *Thermus thermophilus* Can1 (TtCan1) nuclease activities.** Cleavage assays with TtCan1 (200 nM) in the presence of various cyclic oligonucleotides (50 nM) in Mn2+ or Mg2+ containing buffers. Reactions were performed with 15 nM plasmid DNA (**a**), 425 nM ssRNA (**b**) or 15 nM ssDNA (**c**), for 15 min at 60°C. Reactions with plasmid DNA were stopped by adding Gel Loading Dye, Purple (6X) (NEB) and visualized in 1% agarose gel. For ssDNA and ssRNA cleavage assays, 2X RNA Loading Dye (NEB) was added, and 10 µL was used for 12% UREA PAGE.

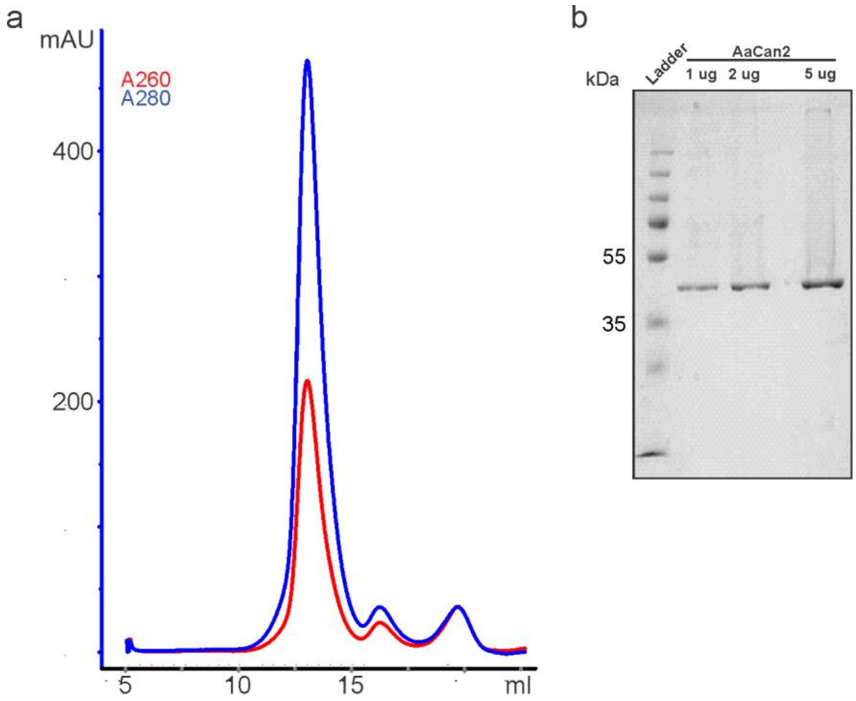


**Supplementary Figure 3: Purification of Can2 from *Archaeoglobi* archaeon JdFR-42. a** Size-exclusion chromatography (SEC) of *Archaeoglobi* archaeon Can2 protein (AaCan2). SEC was performed using a Superdex 200 10/300 GL size-exclusion column (Cytiva). **b** SDS-PAGE of purified AaCan2 in three different concentrations. Expected molecular weight is 42.8 kDa.

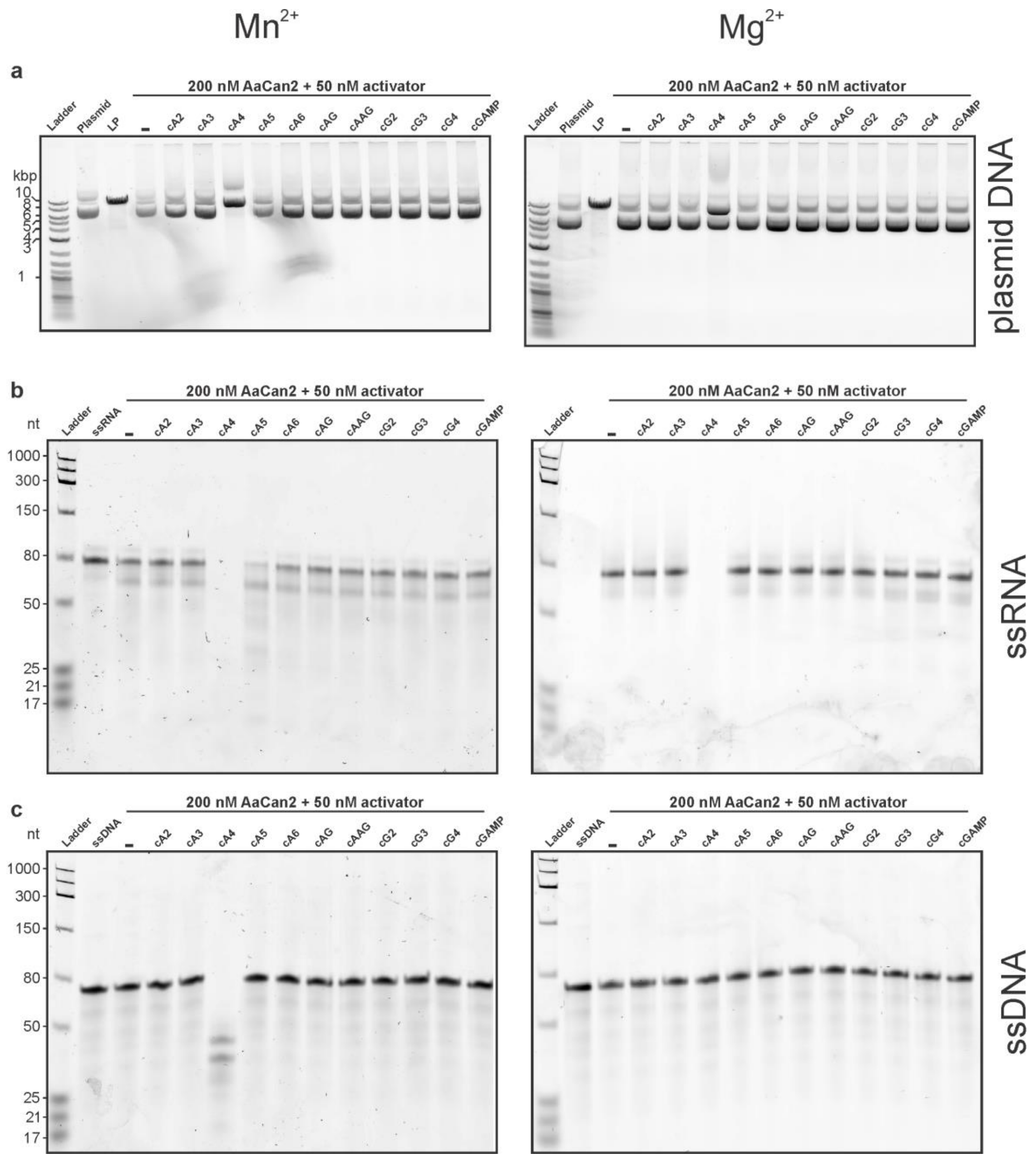


**Supplementary Figure 4: Biochemical characterization of Can2 from *Archaeoglobi* archaeon JdFR-42.** Cleavage assays with AaCan2 (200 nM) in the presence of various cyclic oligonucleotides (50 nM) in Mn2+ or Mg2+ containing buffers. Assays were performed with 15 nM plasmid DNA (**a**), 425 nM ssRNA (**b**) or 15 nM ssDNA (**c**), for 15 min at 60°C. Reactions with plasmid DNA were stopped by adding Gel Loading Dye, Purple (6X) (NEB) and were loaded on 1% agarose gel. For ssDNA and ssRNA cleavage assays, 2X RNA Loading Dye (NEB) was added and 10 µL was loaded on 12% UREA PAGE.

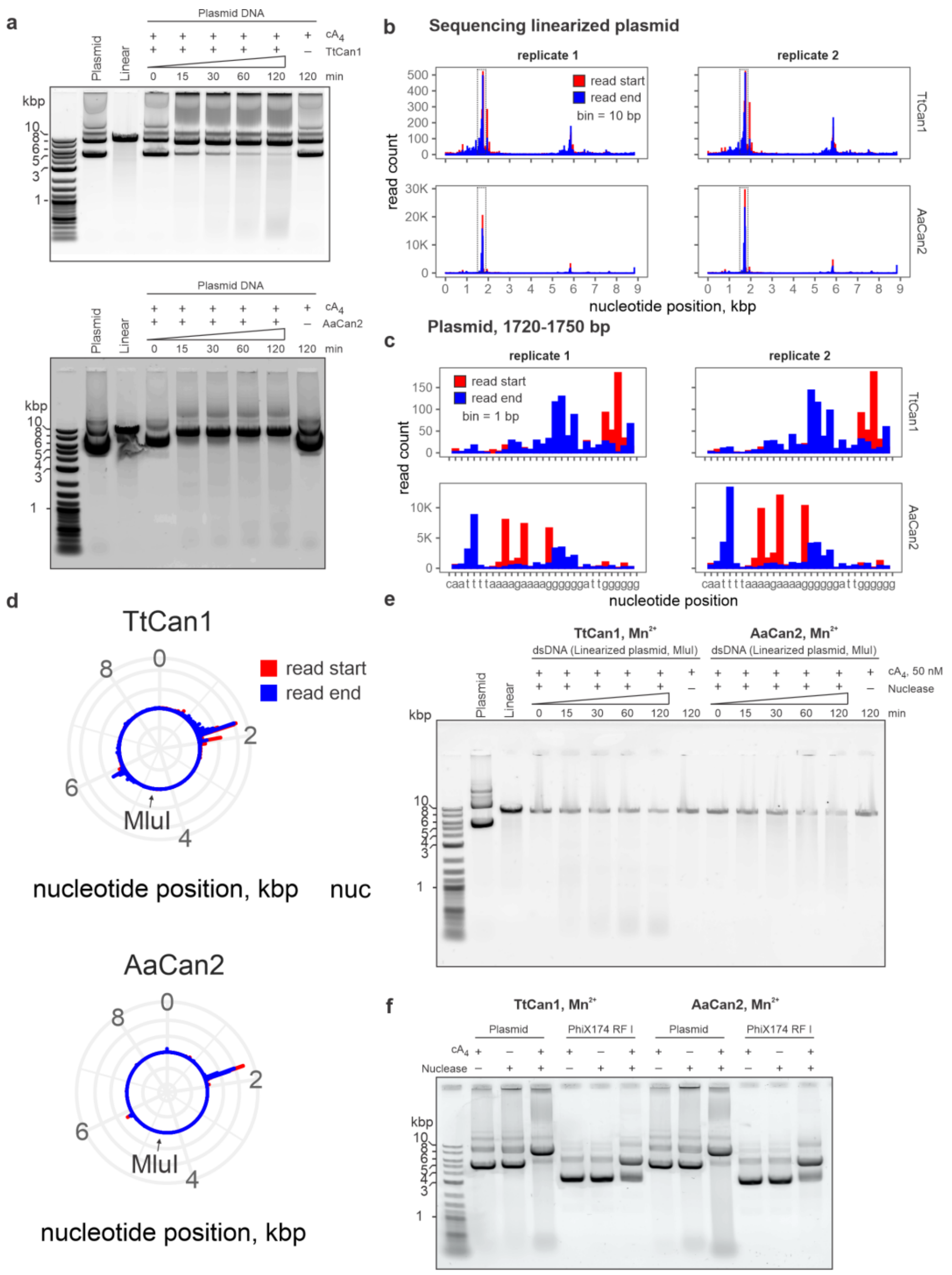


**Supplementary Figure 5: TtCan1 and AaCan2 linearize plasmid DNA in unwound regions. a** Time-course of plasmid DNA (Addgene #105621) incubated with TtCan1 (top, 200 nM) and AaCan2 (bottom, 200 nM) in the presence of $cA_4$ (50 nM). **b** Plasmid DNA (Addgene #105621, 8,833 bp) was incubated with TtCan1 or AaCan2 in the presence of $cA_4$ for 15 min. DNA was purified using Clean-up and Concentration kit (Zymo Research), end-repaired and sequenced using Oxford Nanopore platform. Sequencing reads (≥8,800) were mapped to the reference sequence. Start and end positions of each read were extracted with *bedtools* and plotted with *ggplot*. Bars show number of reads that start (red) or end (blue) at the specific nucleotide position (bin width = 10 bp). **c** Region with the highest cleavage frequency (1,720-1,750 bp; dotted box in **b**) is shown. **d - e** Plasmid DNA (Addgene #105621) was linearized using MluI restriction nuclease, purified with DNA Clean-up and Concentration kit (Zymo Research), incubated with TtCan1 (200 nM) or AaCan2 (200 nM) in the presence of $cA_4$ for 0, 15, 30 60 or 120 min, and visualized using agarose gel electrophoresis. **f** Plasmid DNA (Addgene, #105621) or the replicative form (RF I, NEB N3021) of ΦX174 bacteriophage DNA (which is supercoiled double-stranded DNA) were incubated with TtCan1 (200 nM, left) or AaCan2 (200 nM, right) in the presence of $cA_4$ (50 nM) for 15 min.

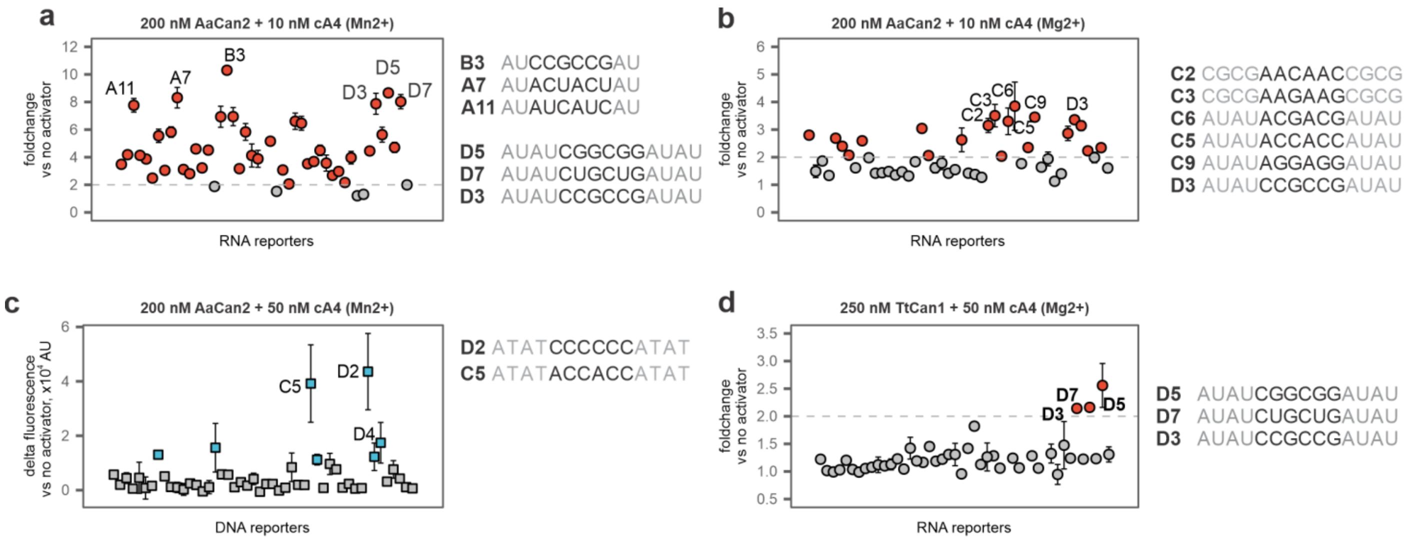


**Supplementary Figure 6: Sequence cleavage preference for AaCan2 and TtCan1.** A library of 46 synthetic RNAs or DNAs tethered to a 5'fluorophore and 3'quencher (see Methods) was screened with AaCan2 (**a, b, c**) and TtCan1 (**d**, only RNA reporters) in the presence of $cA_4$. Reactions were incubated at 55°C in a qPCR instrument with fluorescent readings taken every 10 sec. Fluorescence at 10 min incubation time was normalized to the no activator control. Data are shown as mean ± S.D (n=3).

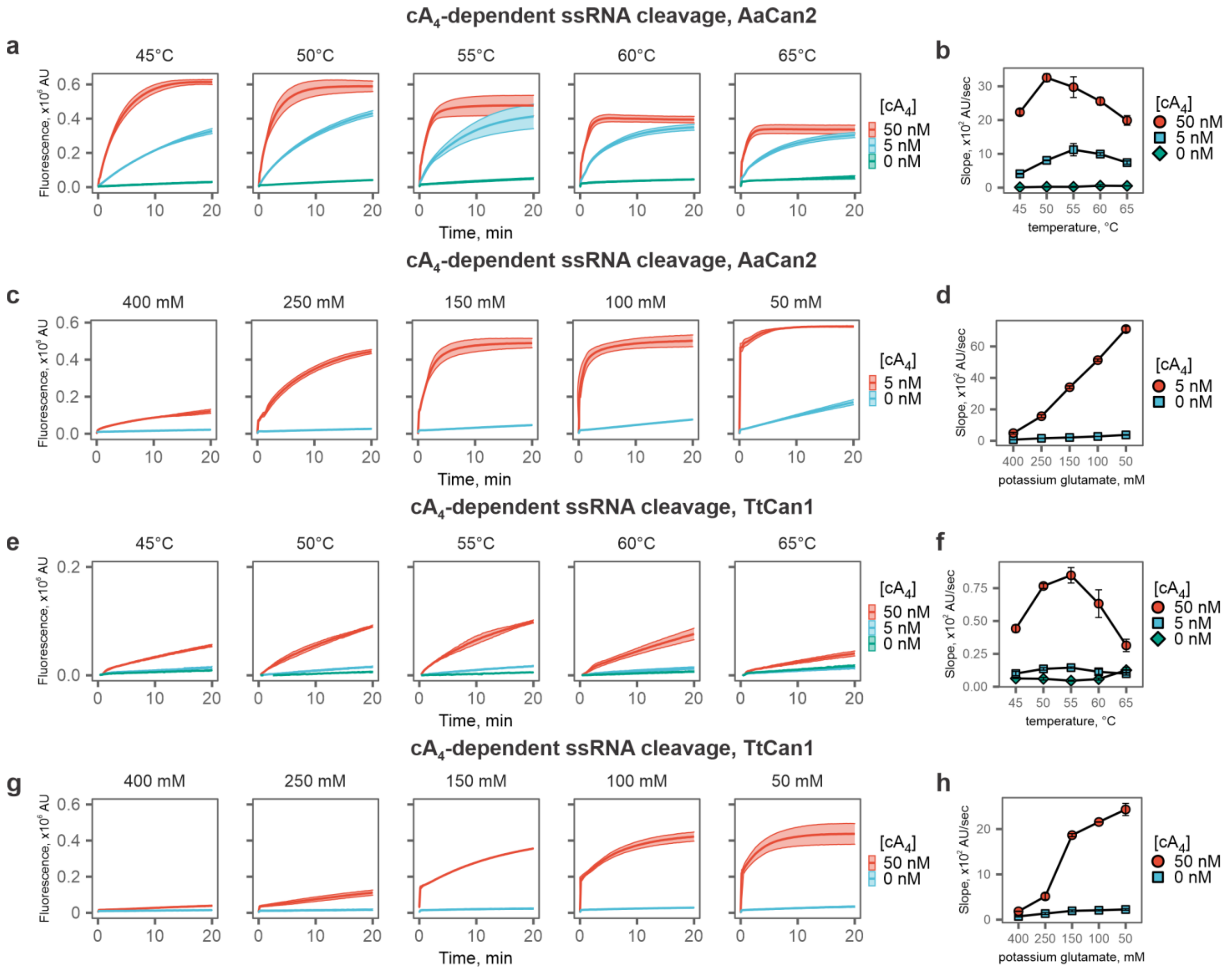


**Supplementary Figure 7: TtCan1 and AaCan2 nuclease activities at different temperatures and salt concentrations.** (**a – d**) AaCan2 (300 nM) and (**e – f**) TtCan1 (300 nM) cleavage assays with ssRNA fluorescent reporter (300 nM, D7 see Supplementary Data 1) in the presence $cA_4$ activator (0, 5 or 50 nM) at different temperatures (45-65°C, 250 mM monopotassium glutamate) or salt concentrations (50-400 mM monopotassium glutamate, 55°C). Data is shown as mean (center line) of two replicates ± S.D. (ribbon) (**a, c**). Simple linear regression was used to calculate slopes for linear regions of the curves (**b, d**). Dots show mean values (n = 2) ± S.D.

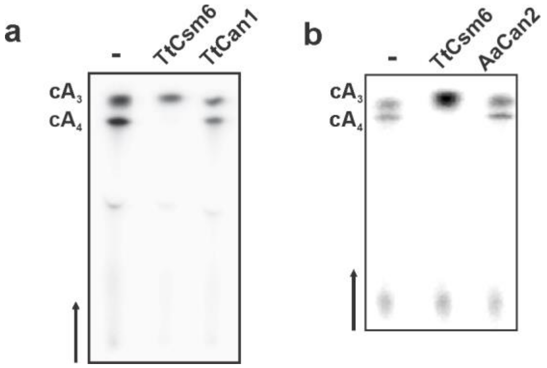


**Supplementary Figure 8: TtCan1 and AaCan2 do not degrade $cA_3$ or $cA_4$.** α-$^{32}$P-labeled $cA_3$ and $cA_4$ were produced using TtCsm$^{Csm3\text{-}D34A}$ complex in the presence of target RNA. Radiolabeled $cA_3$ and $cA_4$ were extracted using phenol-chloroform and mixed with TtCsm6, TtCan1 (500 nM) (**a**) or AaCan2 (500 nM) (**b**) and incubated for 1 hour at 55°C. Reaction products were phenol-chloroform extracted and resolved using thin-layer chromatography (TLC). TtCsm6 cleaves $cA_4$. TtCan1 and AaCan2 degrade neither $cA_3$ nor $cA_4$.

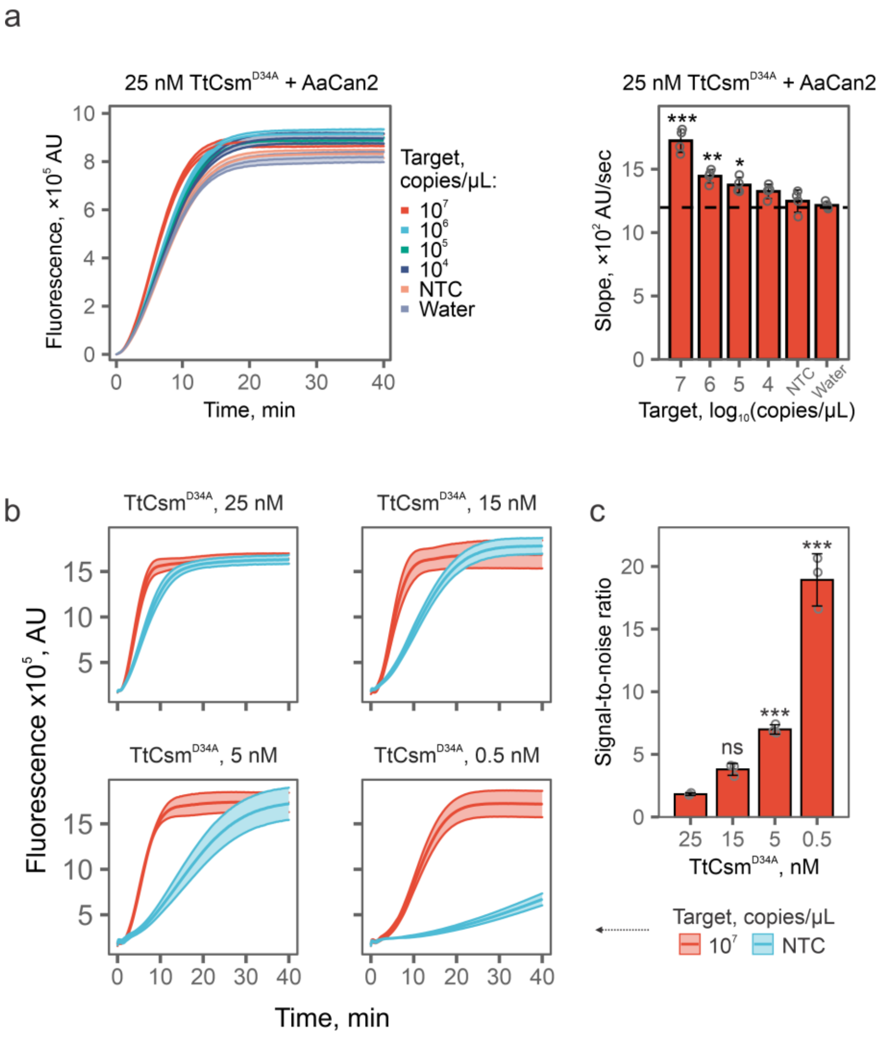


**Supplementary Figure 9: Decreasing TtCsm-complex concentration in RNA detection assay reduces the background signal. a** TtCsm RNA detection assays coupled with AaCan2-based fluorometric detection were performed using samples with target RNA concentrations ranging from $10^7$ to $10^2$ copies/µL. Samples were prepared by spiking IVT fragments of the SARS-CoV-2 N gene into total RNA extracted from a clinical sample that was negative for SARS-CoV-2 by qRT-PCR. Cleavage of the fluorescent RNA reporter was detected by measuring fluorescence every 10 sec in a real-time PCR instrument. *Left:* Data were plotted as mean (centerline) of 4 replicates ± S.D. (ribbon). *Right:* Simple linear regression was used to calculate slopes for linear regions of the curves (at 10 min). Bars show mean values (n = 4) ± S.D. (right). Data was analyzed with one-way ANOVA followed by multiple comparisons to the NTC sample using one-tailed post-hoc Dunnett's test. *** $p < 0.001$; ** $p < 0.01$; * $p < 0.05$. **b** Reactions were performed as described in **a** with varying TtCsm-complex concentrations (25 nM – 0.5 nM). Cleavage of fluorescent RNA reporter was detected by measuring fluorescence every 10 sec in a real-time PCR instrument. Data is shown as mean (center line) of three replicates ± S.D. (ribbon). **c** Signal-to-noise ratios (y-axis) were quantified by dividing slope of fluorescence curves in the assays with target RNA (n= 3, shown in **b**) by mean (n = 3) slope of no target control (NTC). Data is plotted as mean (n = 3) ± S.D. and analyzed with one-way ANOVA. All samples were compared to the reaction with 25 nM $TtCsm^{D34A}$ using two-sided post-hoc Dunnett's test. *** $p < 0.001$; ns – non-significant, $p > 0.05$.

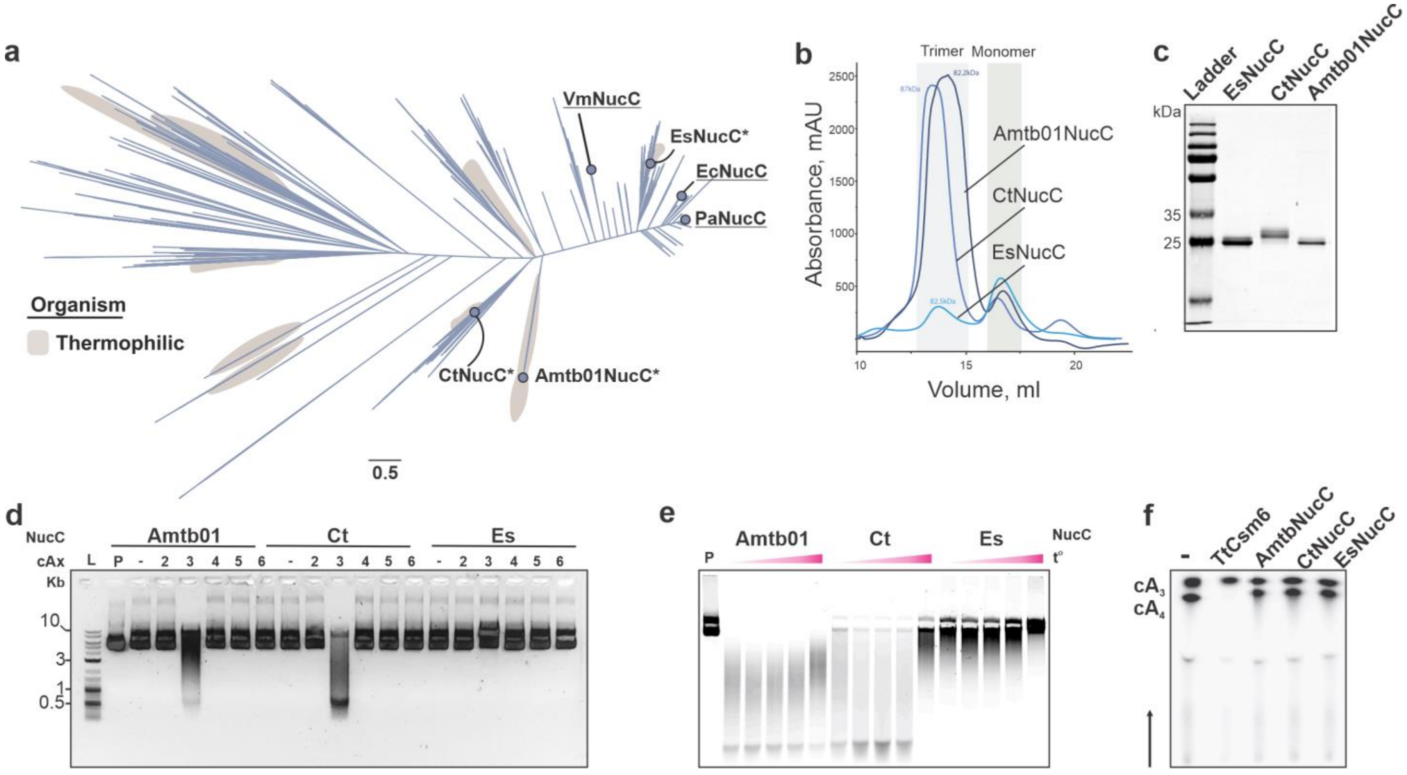


**Supplementary Figure 10: Purification and biochemical characterization of thermostable NucC orthologs. a** Maximum-likelihood phylogeny of 1,510 NucC orthologs. Previously studied effectors are underlined on the tree. Asterisks (*) identify effectors chosen for purification and *in vitro* experiments. **b** Size-exclusion chromatography (SEC) profiles of purified NucC orthologs. SEC was performed using a Sepharose 200 Increase 10/300 GL size-exclusion column (Cytiva). **c** SDS-PAGE of purified NucC orthologs. Expected molecular weight of EsNucC, CtNucC and Amtb01NucC are ~27.5kDA, ~29kDa and ~27.4kDa, respectively. **d** Plasmid DNA (Addgene #105621, 1 µg) digested with thermostable NucC orthologs (45 nM) in the presence of cyclic oligoadenylates with 2–6 bases ($cA_2$-$cA_6$, 45nM). L is the DNA ladder, P is plasmid DNA. **e** DNase activity of NucC orthologs was tested across a temperature range (45-60°C). The plasmid (1 µg) was mixed with 45 nM EsNucC + 45 nM $cA_3$, 15 nM CtNucC + 15 nM $cA_3$ or 45nM Amtb01NucC + 45 nM $cA_3$ for 15 min. **f** $cA_3$ and $cA_4$ hydrolysis assay for EsNucC, CtNucC and Amtb01NucC. The assay was performed as described in Supplementary Figure 8.

35

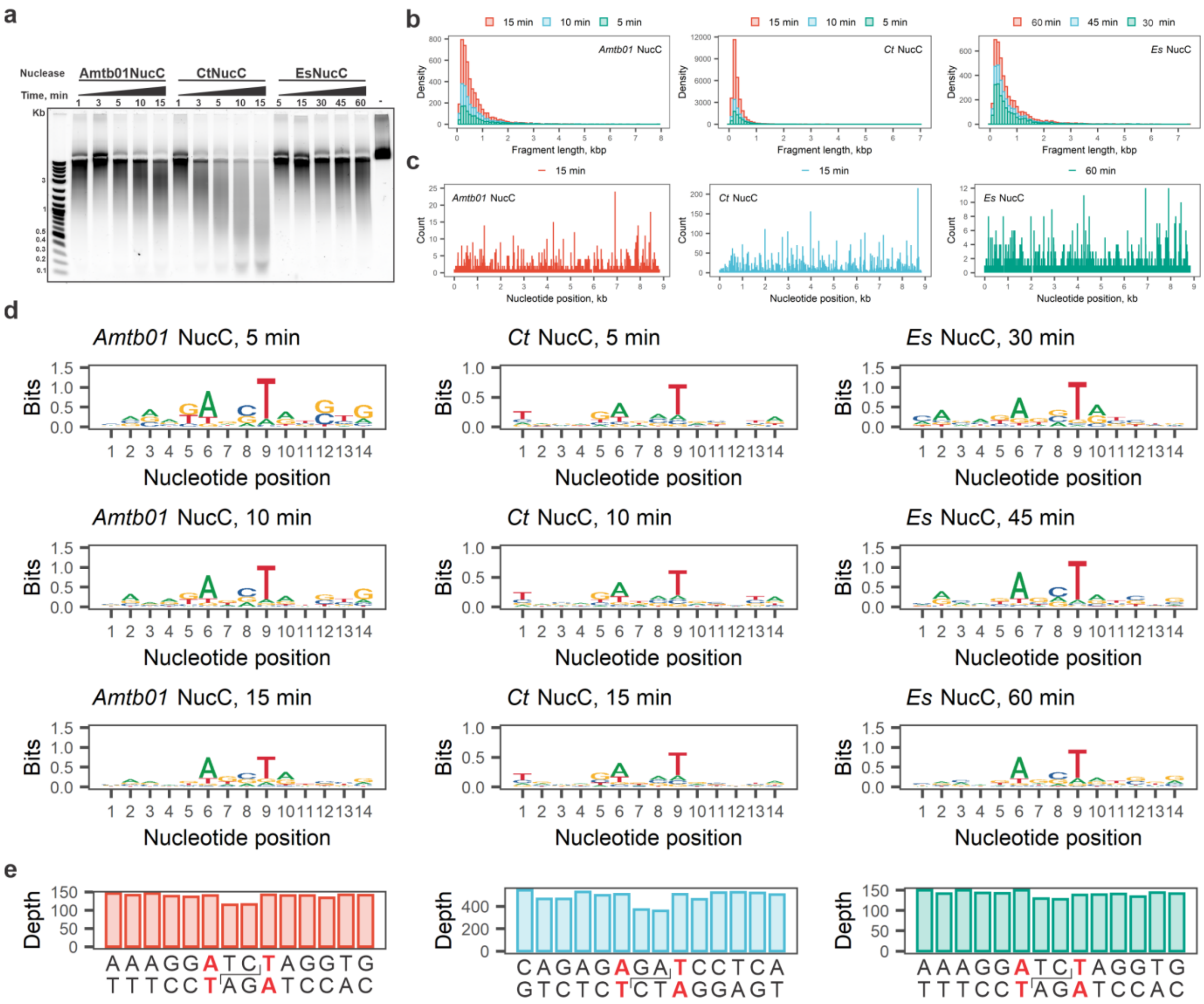


**Supplementary Figure 11: Thermostable NucC homologs Amtb01NucC, EsNucC, and CtNucC demonstrate sequence cleavage preference of plasmid DNA. a** Time-course plasmid DNA (Addgene #105621) digestion with thermostable NucC homologs in the presence of $cA_3$. The plasmid (1 µg) was mixed with EsNucC (45 nM) + $cA_3$ (45 nM), CtNucC (15 nM) + $cA_3$ (15 nM) or Amtb01NucC (45 nM) + $cA_3$ (45 nM) for different periods of time. **b** Plasmid DNA cleavage fragments shown in **a** were end-prepped and deep-sequenced using Oxford Nanopore. Plots show distribution of the read length generated from fragments after different digestion times. **c** Position of read ends (i.e., cut sites) were identified and counted to generate cleavage maps for each nuclease. Resulting maps show cut sites at the longest incubation time tested:15 min of digestion with Amtb01NucC and CtNucC, and after one hour – with EsNucC. **d** Seven nucleotide sequences from both ends of the 5% most frequently cut sites were extracted and used to generate sequence logos. Resulting logos indicate enrichment for A at -1 position (nucleotide 6) and T at +1 position (nucleotide 9). **e** Sequences of top cleaved site are shown for each nuclease. Sequencing depth shows a pattern (i.e., reduced sequencing depth at cut site) which is consistent with 3’-end overhang removal during end-prep step with T4 DNA polymerase when sequencing libraries were prepared.

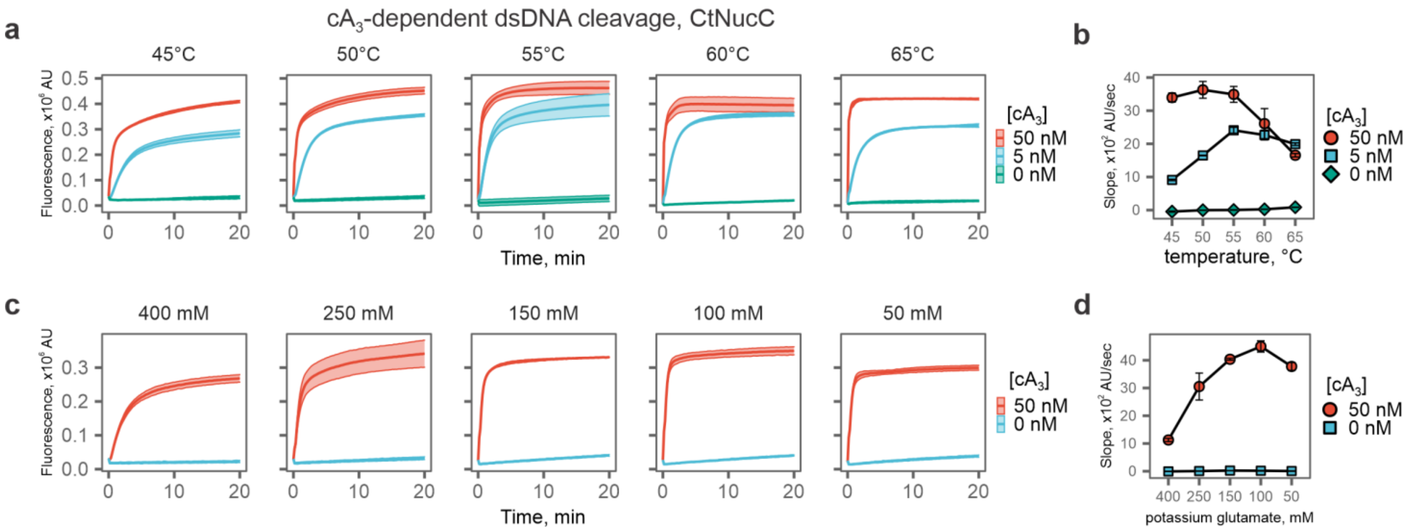


**Supplementary Figure 12: CtNucC nuclease activity at different temperatures and salt concentrations.** CtNucC (300 nM) cleavage assays with fluorescent dsDNA reporter in the presence $cA_3$ activator (5 nM or 50 nM) at different temperatures (45-65°C, 250 mM monopotassium glutamate) (**a, b**) and salt concentrations (50-400 mM monopotassium glutamate, 55°C) (**c, d**). Data is shown as mean (center line) of two replicates ± S.D. (ribbon) (**a, c**). Simple linear regression was used to calculate slopes for linear regions of the curves (**b, d**). Dots show mean values (n = 2) ± S.E.M.

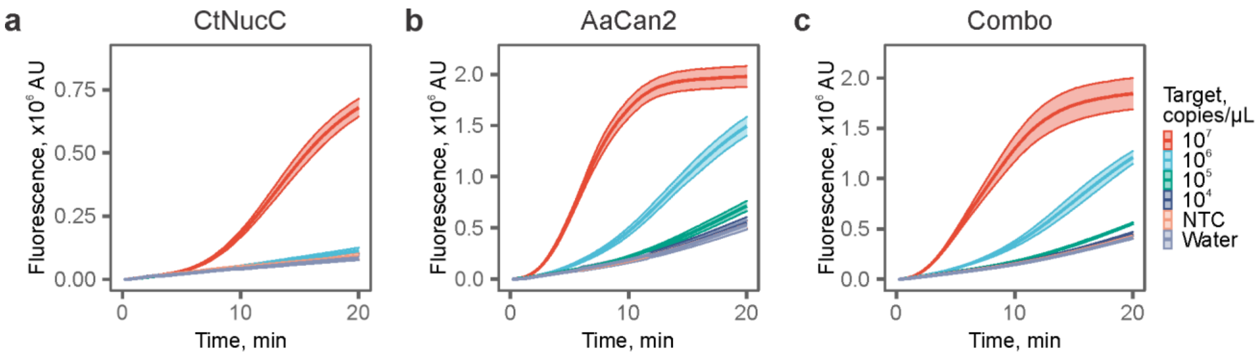


**Supplementary Figure 13: Integration of $cA_3$-activated nucleases into Csm-based RNA detection assay does not improve sensitivity. a** TtCsm RNA detection assays coupled with AaCan2 (ssRNA reporter), CtNucC (dsDNA reporter) and combination of AaCan2 and CtNucC (both reporters). Reactions were performed using samples with target RNA concentration ranging from $10^7$ to $10^4$ copies/µL. Samples were prepared by spiking IVT fragment of SARS-CoV-2 N gene in total RNA of SARS-CoV-2 negative nasal swab. Cleavage of the fluorescent reporter was detected by measuring fluorescence every 10 sec in a real-time PCR instrument. Data is shown as mean (center line) of four replicates ± S.D. (ribbon).

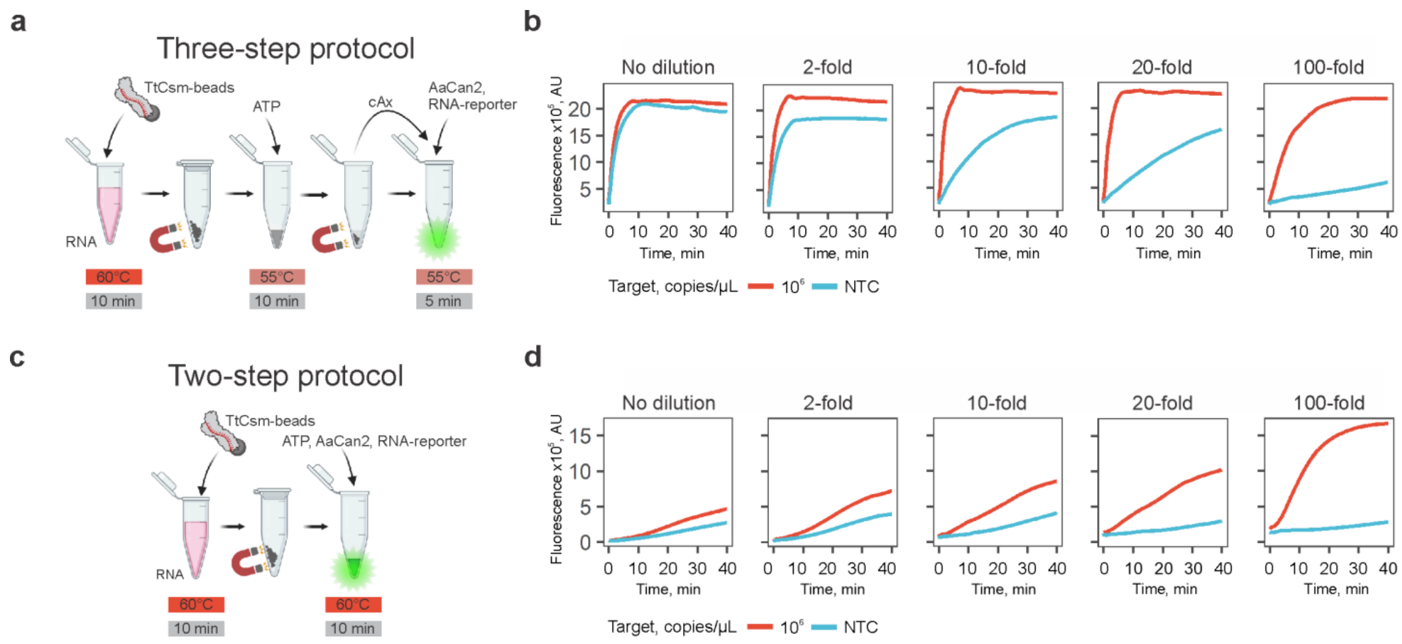


**Supplementary Figure 14: Optimization of TtCsm-based RNA capture and detection assay. a** Three-step protocol includes incubating TtCsm-beads with RNA sample at 60°C for 10 minutes (Step 1), concentrating the beads with a magnet, resuspending the beads in a buffer containing ATP, 10 min incubation (Step 2). Then concentrate the beads again and transferring the polymerization products (supernatant) to a reaction containing AaCan2 and a fluorescent RNA reporter (i.e., FAM-RNA-Iowa Black FQ) (Step 3). **b** Decreasing TtCsm-beads concentration increases signal-to-noise ratio. The reactions were performed as shown in **a** with varying Csm-beads dilutions in the pull-down step (Step 1). SARS-CoV-2 N-gene RNA diluted in total human RNA (HEK 293T cells) was used as the target. Cleavage of fluorescent RNA reporter was detected by measuring fluorescence every 10 sec in a real-time PCR instrument. **c** The two-step protocol includes incubating TtCsm-beads with RNA sample at 60°C for 10 minutes (Step 1), concentrating the beads with a magnet, and one-pot polymerization and fluorescent detection (Step 2). In the Step 2, concentrated TtCsm-beads bound to the target RNA are resuspended in a buffer containing ATP, AaCan2 and a fluorescent RNA reporter (i.e., FAM-RNA-Iowa Black FQ). **d** Decreasing TtCsm-beads concentration rescues target-specific fluorescent signal and increases signal-to-noise ratio. The reactions were performed as shown in **c** with different Csm-beads dilutions in the Step 1. SARS-CoV-2 N-gene RNA diluted in total human RNA (HEK 293T cells) was used as the target. Cleavage of fluorescent RNA reporter was detected by measuring fluorescence every 10 sec in a real-time PCR instrument.

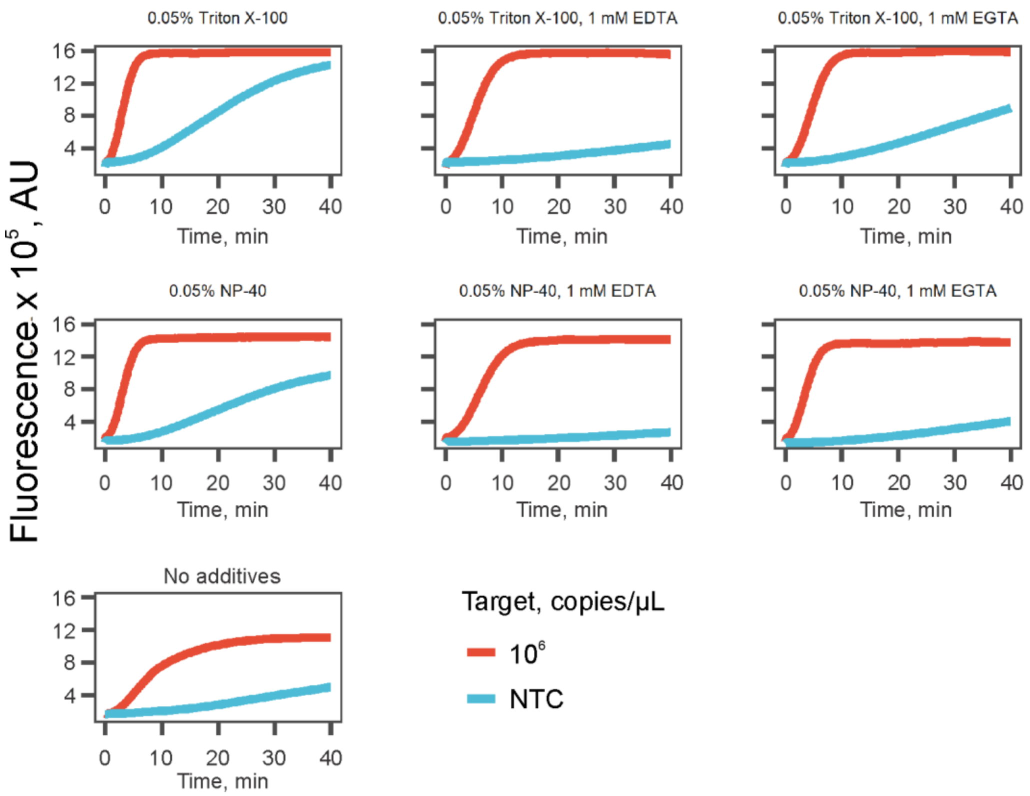


**Supplementary Figure 15: Testing effect of detergents and chelating agents on TtCsm-based RNA capture and detection assay.** Csm-based target RNA concentration followed by one-pot $cA_4$ production (2-step protocol, **Supplementary Fig. 14c**) with fluorescent detection was performed from mock sample containing indicated additives. The mock sample was made by spiking SARS-CoV-2 RNA fragment (final concentration $10^6$ copies/μL) into total human RNA (HEK 293T cells). Total human RNA was used as the no target control (NTC). Cleavage of fluorescent RNA reporter was detected by measuring fluorescence every 10 sec in a real-time PCR instrument.

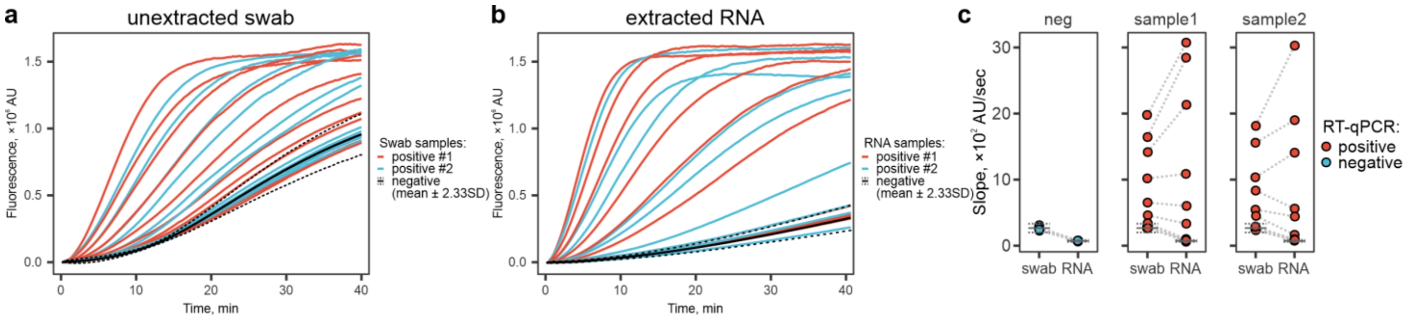


**Supplementary Figure 16: Direct detection of SARS-CoV-2 RNA in clinical samples using TtCsm-based RNA capture. a, b** Two nasopharyngeal swabs were serially diluted in a negative patient swab sample (#1, Ct~14.8, red lines; #2, Ct~15.6, blue lines) and twenty-four dilutions in total were tested with type III RNA capture & detection assay directly (**a**) or from priorly purified RNA (**b**). Black solid line shows mean fluorescence in negative samples (n = 6), dotted black lines show ± 2.33 S.D. interval from mean. **c** Simple linear regression was used to calculate slopes for linear regions of the curves in **a** and **b**. Horizontal dotted lines show mean slope in negative samples (n = 6) ± 2.33 S.D. Reactions that generated signal higher than upper bound of this interval were considered positive for SARS-CoV-2 RNA in the type III CRISPR diagnostic assay. Dashed gray lines connect same samples that were either tested directly with type III RNA capture & detection assay (swab) or extracted (RNA) before type III RNA detection.

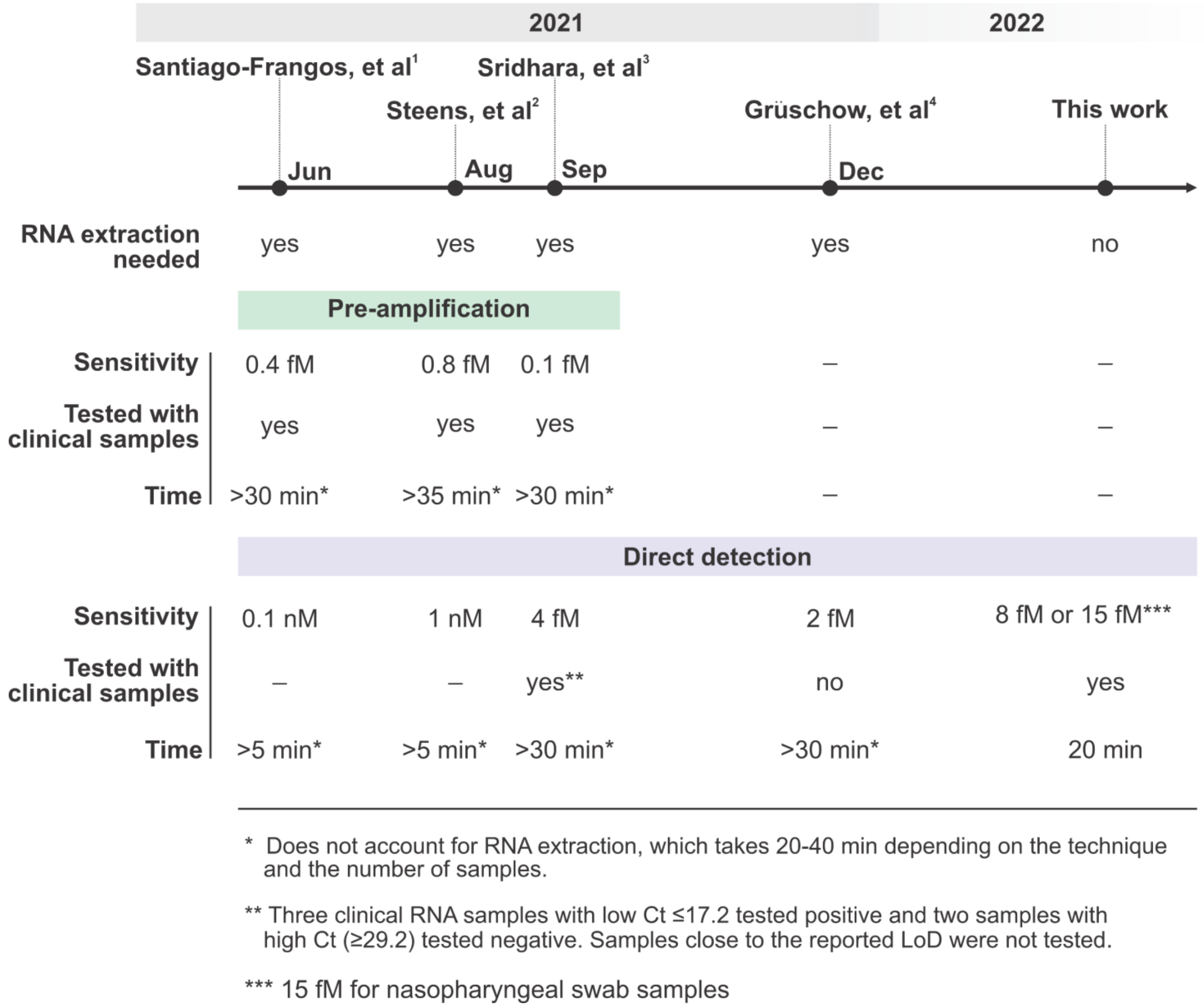


**Supplementary Figure 17: The evolution of type III-based diagnostics.** The first iterations of type III-based diagnostics required RNA extraction and pre-amplification to reach clinically relevant sensitivity. More recent improvements of type-III based diagnostics have been used to detect spiked SARS-CoV-2 RNA with ~2-4 fM sensitivity without pre-amplification. This work demonstrates how type-III based diagnostic detects ~ 8 fM of pure SARS-CoV-2 RNA and ~ 15 fM of SARS-CoV-2 RNA directly in unprocessed patient samples (i.e., nasopharyngeal swabs) in 20 min.

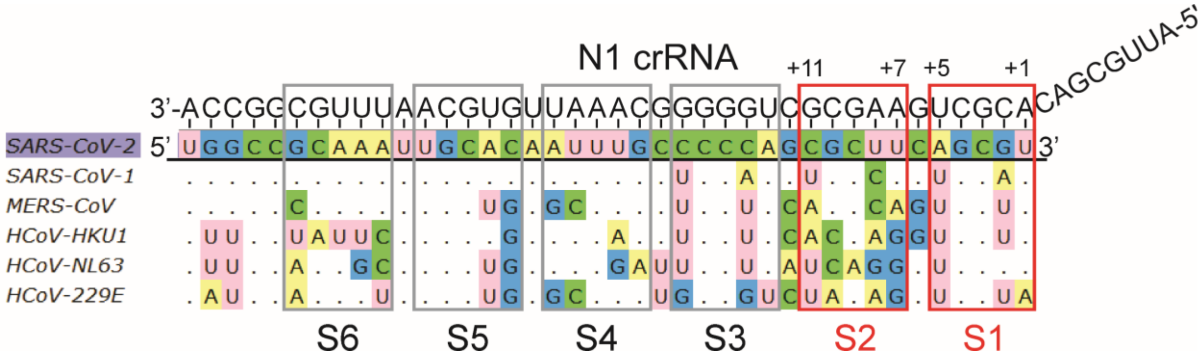


**Supplementary Figure 18:** Alignment of N gene sequences targeted by TtCsm-complex in SARS-CoV-2 and other coronaviruses in the cross-reactivity assay. Matching nucleotides are shown with dots, mismatches with nucleotide symbols. Boxes show segments (S1-6) of the crRNA:target RNA duplex bound by backbone subunits of the Csm-complex. Mismatches in S1 and S2 (red) permit RNA binding but significantly reduce ATP polymerization by Cas10 subunits.

**Supplementary Table 1.** RNase P RNA levels in nasopharyngeal swab samples and diluted total RNA from HEK 293T cells used as negative control in the manuscript.

| Sample | Ct value (RP primers, 2019-nCoV CDC EUA Kit, IDT#10006606) |
|---|---|
| RNA patient sample 1 | 26.85 |
| RNA patient sample 2 | 24.73 |
| RNA patient sample 3 | 35.41 |
| RNA patient sample 4 | 28.29 |
| RNA patient sample 5 | 28.58 |
| RNA patient sample 6 | 20.36 |
| RNA patient sample 7 | 25.24 |
| RNA patient sample 8 | 25.14 |
| RNA patient sample 9 | 28.15 |
| RNA patient sample 10 | 30.37 |
| Total RNA from HEK 293T (diluted) | 23.35 |

**Supplementary Table 2.** Oligonucleotide primers used to generate DNA templates for in vitro transcription with T7 polymerase

| Primer name | Sequence (5’-3’) |
|---|---|
| SARS-CoV-2 N1 T7 template Forward | GATAATACGACTCACTATAGGGAACTGATTACAAACATTGGCCGCAAATTGCA CAATT |
| SARS-CoV-2 N1 T7 template Reverse | GCGCGACATTCCGAAGAACGCTGAAGCGCTGGGGGCAAATTGTGCAATTTGCG GCC |
| SARS-CoV-1 N1 T7 template Forward | GATAATACGACTCACTATAGGGAACTGATTACAAACATTGGCCGCAAATTGCA CAATT |
| SARS-CoV-1 N1 T7 template Reverse | GCGTGACATTCCAAAGAATGCAGAGGCACTTGGAGCAAATTGTGCAATTTGCG GCC |

## Supplementary References

1. Santiago-Frangos, A., Hall, L.N., Nemudraia, A., Nemudryi, A., Krishna, P., Wiegand, T., Wilkinson, R.A., Snyder, D.T., Hedges, J.F., and Cicha, C. (2021). Intrinsic signal amplification by type III CRISPR-Cas systems provides a sequence-specific SARS-CoV-2 diagnostic. Cell Reports Medicine *2*, 100319. DOI: 10.1016/j.xcrm.2021.100319

2. Steens, J.A., Zhu, Y., Taylor, D.W., Bravo, J.P.K., Prinsen, S.H.P., Schoen, C.D., Keijser, B.J.F., Ossendrijver, M., Hofstra, L.M., Brouns, S.J.J., et al. (2021). SCOPE enables type III CRISPR-Cas diagnostics using flexible targeting and stringent CARF ribonuclease activation. Nature Communications *12*. DOI: 10.1038/s41467-021-25337-5.

3. Sridhara, S., Goswami, H.N., Whyms, C., Dennis, J.H., and Li, H. (2021). Virus detection via programmable Type III-A CRISPR-Cas systems. Nature Communications *12*. DOI: 10.1038/s41467-021-25977-7.

4. Grüschow, S., Adamson, C.S., and White, M.F. (2021). Specificity and sensitivity of an RNA targeting type III CRISPR complex coupled with a NucC endonuclease effector. Nucleic Acids Research *49*, 13122-13134. DOI: 10.1093/nar/gkab1190.
