# Supplementary material for: Sequence-specific capture and concentration of viral RNA by type III CRISPR system enhances diagnostic
Source: Nat Commun. 2022 Dec 15;13:7762. doi: 10.1038/s41467-022-35445-5 (PMC9751510; doi:10.1038/s41467-022-35445-5)
Supplement: Supplementary file 3 — Description of Additional Supplementary Files [file 41467_2022_35445_MOESM3_ESM.docx]

**Description of Supporting Information**

**Supplementary Data 1.** Sequences of fluorescent RNA and DNA reporters.
