# Supplementary material for: Sequence-specific capture and concentration of viral RNA by type III CRISPR system enhances diagnostic
Source: Nat Commun. 2022 Dec 15;13:7762. doi: 10.1038/s41467-022-35445-5 (PMC9751510; doi:10.1038/s41467-022-35445-5)
Supplement: Supplementary file 5 — Reporting Summary [file 41467_2022_35445_MOESM5_ESM.pdf]

nature portfolio

Corresponding author(s): Blake Wiedenheft

Last updated by author(s): Nov 22, 2022

Reporting Summary

Nature Portfolio wishes to improve the reproducibility of the work that we publish. This form provides structure for consistency and transparency in reporting. For further information on Nature Portfolio policies, see our Editorial Policies and the Editorial Policy Checklist.

Statistics

For all statistical analyses, confirm that the following items are present in the figure legend, table legend, main text, or Methods section.

n/a	Confirmed	
☐	☒	The exact sample size (*n*) for each experimental group/condition, given as a discrete number and unit of measurement
☐	☒	A statement on whether measurements were taken from distinct samples or whether the same sample was measured repeatedly
☐	☒	The statistical test(s) used AND whether they are one- or two-sided<br>*Only common tests should be described solely by name; describe more complex techniques in the Methods section.*
☒	☐	A description of all covariates tested
☒	☐	A description of any assumptions or corrections, such as tests of normality and adjustment for multiple comparisons
☐	☒	A full description of the statistical parameters including central tendency (e.g. means) or other basic estimates (e.g. regression coefficient) AND variation (e.g. standard deviation) or associated estimates of uncertainty (e.g. confidence intervals)
☐	☒	For null hypothesis testing, the test statistic (e.g. *F*, *t*, *r*) with confidence intervals, effect sizes, degrees of freedom and *P* value noted<br>*Give P values as exact values whenever suitable.*
☒	☐	For Bayesian analysis, information on the choice of priors and Markov chain Monte Carlo settings
☒	☐	For hierarchical and complex designs, identification of the appropriate level for tests and full reporting of outcomes
☒	☐	Estimates of effect sizes (e.g. Cohen's *d*, Pearson's *r*), indicating how they were calculated

Our web collection on statistics for biologists contains articles on many of the points above.

Software and code

Policy information about availability of computer code

Data collection	Gels were scanned with Typhoon 5 imager (Amersham). The fluorescence measurements were performed using a real-time PCR instrument QuantStudio 3 (Applied Biosystems). Nanopore sequencing data was collected with MinION Mk1B device (MIN-101B, R9.4.1 flow cells). Basecalling was performed in the high-accuracy mode using MinKNOW software v5.2.4.
Data analysis	Gel bands were quantified using ImageJ v1.52t. Statistical testing, phylogenetic analysis and data plotting was performed using publicly available software packages that are described in the Methods section. The manuscript haven't produced any new software. Code used to perform phylogenetic analyses, analyze sequencing data and design reporter libraries is available through Wiedenheft lab GitHub webpage and/or Zenodo repository.

For manuscripts utilizing custom algorithms or software that are central to the research but not yet described in published literature, software must be made available to editors and reviewers. We strongly encourage code deposition in a community repository (e.g. GitHub). See the Nature Portfolio guidelines for submitting code & software for further information.

Data

Policy information about availability of data

All manuscripts must include a data availability statement. This statement should provide the following information, where applicable:

- Accession codes, unique identifiers, or web links for publicly available datasets
- A description of any restrictions on data availability
- For clinical datasets or third party data, please ensure that the statement adheres to our policy

The Nanopore sequencing data have been deposited to the Zenodo repository (DOI: 10.5281/zenodo.7374621). Phylogenetic analyses data generated in the current study are available on Wiedenheft lab GitHub page (https://github.com/WiedenheftLab/; DOI: 10.5281/zenodo.7368902 , 10.5281/zenodo.7369225). Source data are provided with this paper.

Human research participants

Policy information about studies involving human research participants and Sex and Gender in Research.

Reporting on sex and gender	Study uses de-identified samples collected from COVID-19 patients. No data on sex and gender was collected.
Population characteristics	See above
Recruitment	Clinical samples were obtained with informed consent from patients undergoing testing for SARS-CoV-2 at Bozeman Health Deaconess Hospital.
Ethics oversight	Montana State University Institutional Review Board (IRB) For the Protection of Human Subjects (FWA 00000165)

Note that full information on the approval of the study protocol must also be provided in the manuscript.

Field-specific reporting

Please select the one below that is the best fit for your research. If you are not sure, read the appropriate sections before making your selection.

[x] Life sciences [ ] Behavioural & social sciences [ ] Ecological, evolutionary & environmental sciences

For a reference copy of the document with all sections, see nature.com/documents/nr-reporting-summary-flat.pdf

Life sciences study design

All studies must disclose on these points even when the disclosure is negative.

Sample size	All experiments were performed in three replicates (unless stated otherwise), which is standard for such experimental designs. No prior sample size calculation was performed.
Data exclusions	No data were excluded from the analyses.
Replication	All experiments were performed in multiple replicates (3 or more). To verify reproducibility described nuclease activities, multiple independent protein purifications were performed by multiple lab members. RT-qPCR experiments were performed in three technical and three biological replicates. All reported results were reproducible.
Randomization	Randomization was not performed in bionformatic and biochemical experiments. Biochemical reactions were performed identically and measured at the same time in a qPCR intsrument to make direct comparisons to a control.
Blinding	Type III based RNA detection and qPCR experiments were performed independently on the same set of clinical samples. Investigators were not blinded during data collection or analysis. Direct detection in swabs was performed using serial dilutions of a sample with low Ct value and was not blinded. No blinding was performed in the biochemical assays because it was necessary to know exact composition of each reaction to characterize nuclease activities and determine sensitivity and specificity of the diagnostic assay.

Reporting for specific materials, systems and methods

We require information from authors about some types of materials, experimental systems and methods used in many studies. Here, indicate whether each material, system or method listed is relevant to your study. If you are not sure if a list item applies to your research, read the appropriate section before selecting a response.

Materials & experimental systems

n/a	Involved in the study
☒	☐ Antibodies
☐	☒ Eukaryotic cell lines
☒	☐ Palaeontology and archaeology
☒	☐ Animals and other organisms
☒	☐ Clinical data
☒	☐ Dual use research of concern

Methods

n/a	Involved in the study
☒	☐ ChIP-seq
☒	☐ Flow cytometry
☒	☐ MRI-based neuroimaging

Eukaryotic cell lines

Policy information about cell lines and Sex and Gender in Research

Cell line source(s)	The study uses total RNA extracted from HEK 293T cells. The cell line was obtained from ATCC (Cat. No. CRL-3216)
Authentication	293T cells were authenticated by ATCC.
Mycoplasma contamination	Cell are routinely tested for contamination using PCR with oligonucleotide primers targeting Mycoplasma genome. RNA was extracted from cells that previously tested negative.
Commonly misidentified lines (See ICLAC register)	Commonly misidentified lines were not used.
